# Role of diffusion-weighted imaging in the discrimination of benign and metastatic parotid area lymph nodes in patients with nasopharyngeal carcinoma

**DOI:** 10.1038/s41598-017-18617-y

**Published:** 2018-01-10

**Authors:** Chuanben Chen, Zhizhong Lin, Youping Xiao, Penggang Bai, Qiuyuan Yue, Yunbin Chen, Lisha Chen

**Affiliations:** 1 Department of Radiation Oncology, Fujian Cancer Hospital, Fujian Medical Universtiy Cancer Hospital, Fuzhou, Fujian China; 2 Shengli Clinical Medical College of Fujian Medical University, Fuzhou, Fujian China; 3 Fujian Provincial Key Laboratory of Translational Cancer Medicine, Fuzhou, Fujian China; 4 Department of Radiology, Fujian Cancer Hospital, Fujian Medical Universtiy Cancer Hospital, Fuzhou, Fujian China

## Abstract

To assess the utility of apparent diffusion coefficient (ADC) determined on diffusion-weighted MR imaging (DWI) to differentiate between benign and malignant parotid area lymph nodes (PLN) in nasopharyngeal carcinoma (NPC) patients. Thirty-nine consecutive NPC patients with a total of 40 enlarged, biopsied PLNs underwent DWI examination. ADC values for benign and malignant PLNs were measured and compared. Receiver operating characteristic (ROC) curve analysis was to evaluate the optimal threshold level of ADC values for metastatic PLNs. The *k*appa was to assess the degree of agreement between histopathological diagnoses and ADC values, or imaging features of PLNs on MRI. We found the mean ADC value for benign PLNs was markedly higher than malignant PLNs. A threshold ADC of 1.01 × 10^−3^ mm^2^/s was associated with a sensitivity of 85.7% and a specificity of 72.7% (area under the curve: 0.84). A moderate agreement was observed between the histopathological diagnosis and the threshold of ADC value (*k* value: 0.483). However, short axis diameter, necrosis, extranodal extension, and regional grouping of PLNs on MRI showed only a fair agreement with the histopathological diagnosis (*k* value: 0.257, 0.305, 0.276, and 0.205, respectively). Therefore, DWI may be a promising technique to differentiate metastatic from benign PLNs.

## Introduction

Nasopharyngeal carcinoma (NPC) is often accompanied by metastatic involvement of lymph nodes in the neck region. However, parotid area lymph node (PLN) metastases from NPC are uncommon (estimated incidence: 1% ~3.4%)^1–4^. The current employed radiological criteria for metastatic involvement of PLNs in patients with NPC are mainly based on malignant involvement of cervical lymph nodes, including the evaluation of their size and morphological abnormalities on computed tomography (CT) or magnetic resonance imaging (MRI)^5–8^. In recent years, however, some PLNs which did not qualify the radiological criteria were later found to develop periparotid recurrence, especially with the prevailing practice of parotid gland-sparing intensity-modulated radiotherapy (IMRT)^9–11^. Based on these findings, the radiological criteria for PLNs need to be further investigated. In addition, the PLNs are commonly too small to be amenable to fine-needle aspiration cytology (FNAC) for pathological examination prior to the treatment of NPC. In clinical practice, a minimal axial diameter (MID) of ≥10 mm of PLNs on cross-sectional images is generally accepted as an indication for FNAC. Furthermore, patients tend to be reluctant to undergo FNAC owing to the invasive nature of the procedure unless there is a high suspicion of malignant involvement of PLNs on imaging. Therefore, there is a pressing need to establish novel radiological criteria so as to help clinicians detect metastatic nodes in the parotid glands and to guide the clinical treatment of NPC.

Diffusion-weighted MR imaging (DWI) is on the basis of intravoxel incoherent imaging, which makes it more sensitive to subtle abnormalities, and to provide diagnostic information by different pathological characteristics^12^. Apparent diffusion coefficient (ADC) values determined on DWI have been shown to help differentiate malignant from benign lymph nodes in the context of several cancers. Holzapfel *et al*. reported that use of a threshold of ADC value of 1.25 × 10^−3^ mm^2^/s was able to effectively differentiate benign from malignant lymph nodes in patients with cholangiocarcinoma^13^. Similarly, Fornasa *et al*. found ADC as a useful quantitative parameter for the differential diagnosis of axillary lymph nodes in patients with breast cancer^14^. Further, Sumi *et al*. found DWI was effective in the differentiation of metastatic cervical lymph nodes of size ≥10 mm in patients with head and neck cancers^15^.

With regard to NPC, Jin *et al*. reported that use of a cutoff ADC value of 0.924 × 10^−3^ mm^2^/s helped distinguish metastatic (size: 5 to 10 mm in size) from benign neck lymph nodes in patients with NPC^16^. However, to the best of our knowledge, the value of ADC in the discrimination between reactive and metastatic PLNs in NPC has not been well documented to date. Moreover, patients with PLNs may potentially avoid invasive FNAC before the treatment if DWI technique is found to possess high diagnostic value for PLNs in NPC. It is therefore important to assess the use of ADC values on DWI to improve the diagnostic accuracy with respect to the differentiation of benign and malignant PLNs in patients with NPC.

The aim of the present study was to determine whether the technique of DWI could be used to help distinguish benign from malignant parotid area lymphadenopathy in patients with NPC, and to evaluate the degree of agreement between pathological diagnoses and ADC values, as well as the size, or the morphological features of PLNs on MRI.

## Results

### Diffusion-weighted MR images of the PLNs

**Figure 1:**
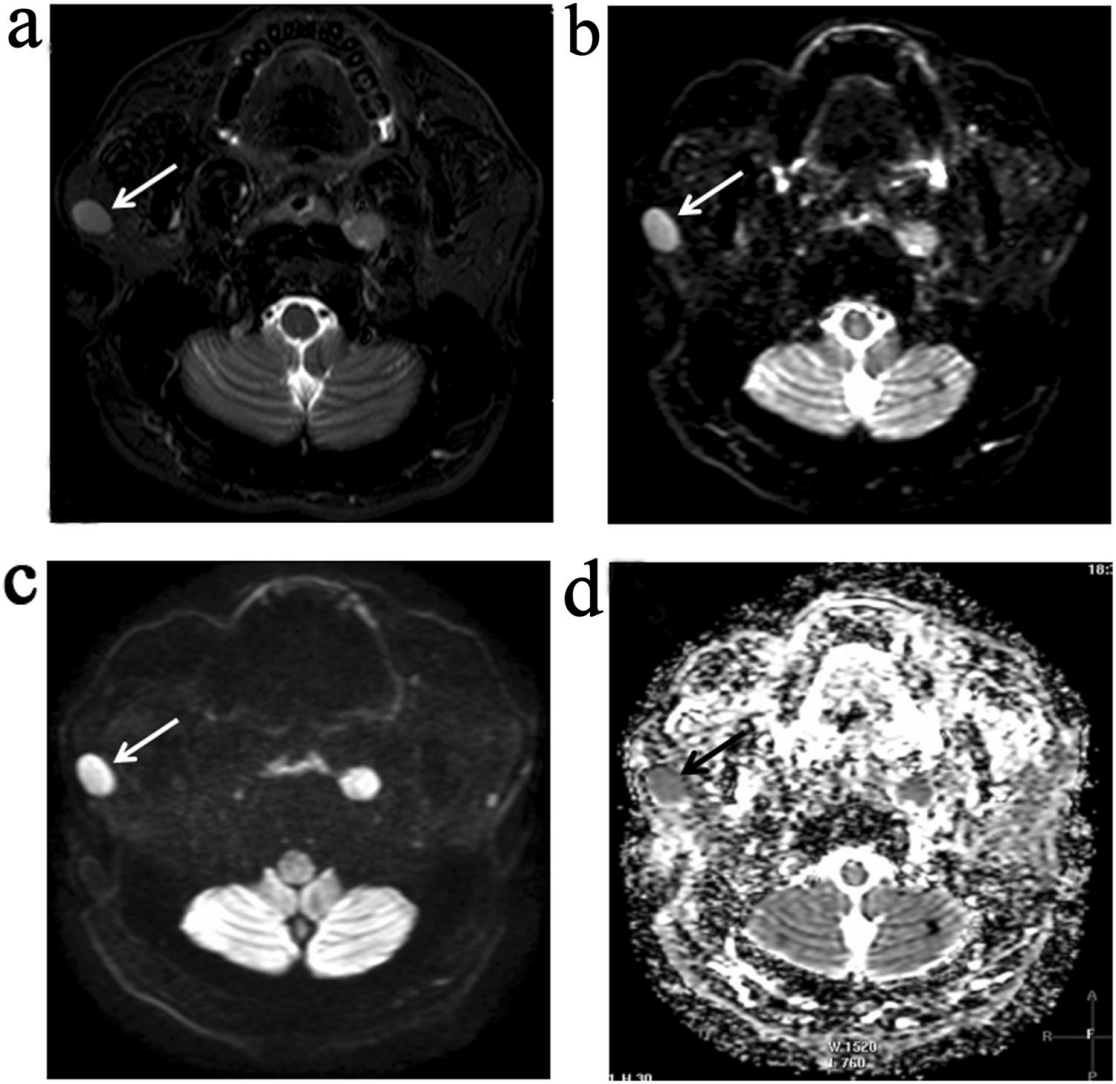
MR images of a metastatic PLN without necrosis (arrows) showing the homogenous signal intensity on a transverse T2-weighted image (**a**). Axial diffusion-weighted MR images show high signals achieved at both *b* = 0 s/mm^2^ (**b**) and *b* = 800 s/mm^2^ (**c**). Consequently, they show low intensity on the ADC map (**d**).

**Figure 2:**
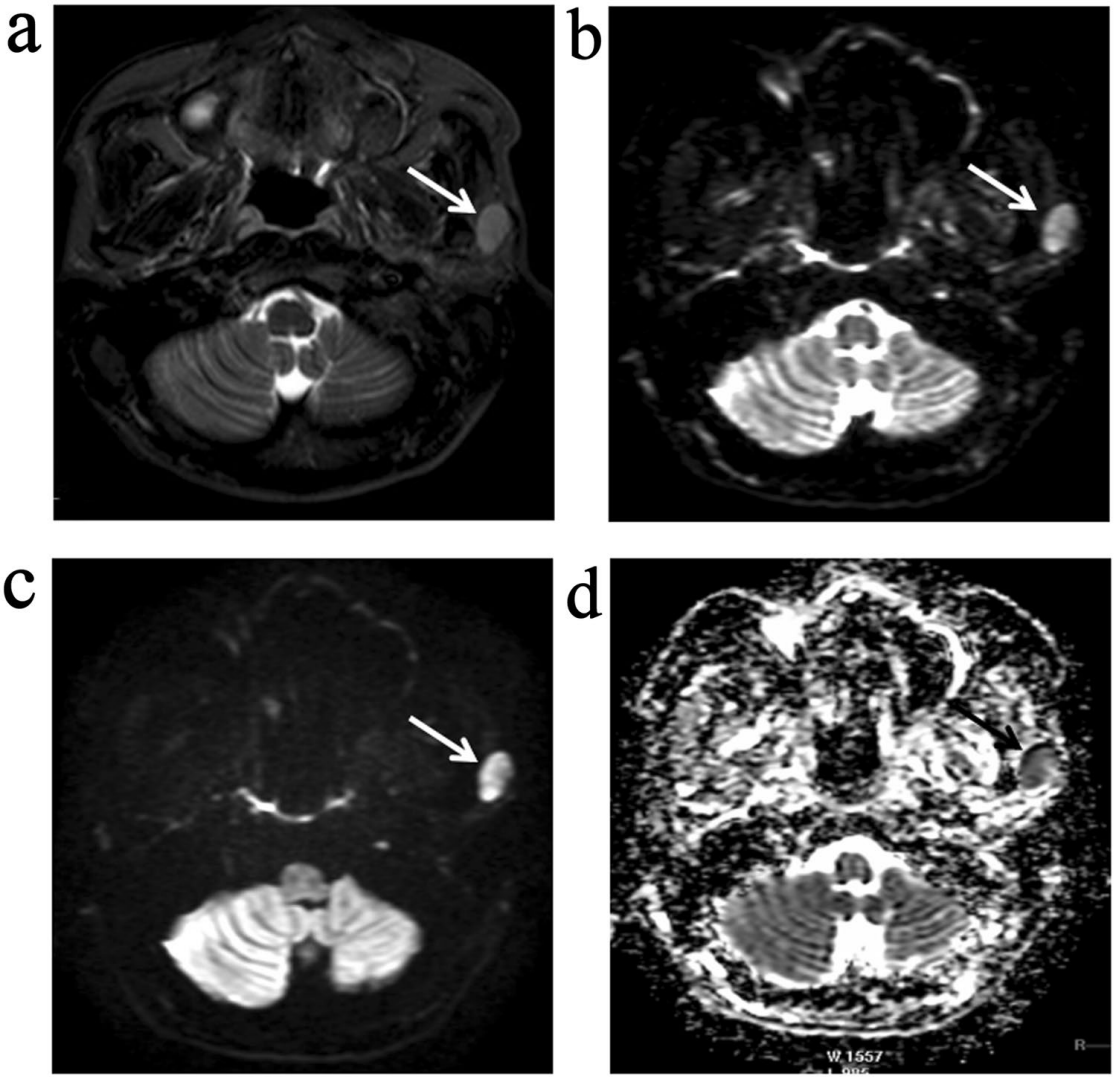
MR images of a metastatic PLN which includes both necrotic and solid portions (arrows) on a transverse T2-weighted image (**a**). Axial diffusion-weighted MR images show heterogeneous signal intensity, including hypointensity of necrotic part at *b* = 0 s/mm^2^ (**b**) and decreased signal intensities at *b* = 800 s/mm^2^ (**c**) compared to the solid components of the malignant PLN. In contrast with solid portions, the necrotic areas show hyperintensity on the ADC map (**d**).

**Figure 3:**
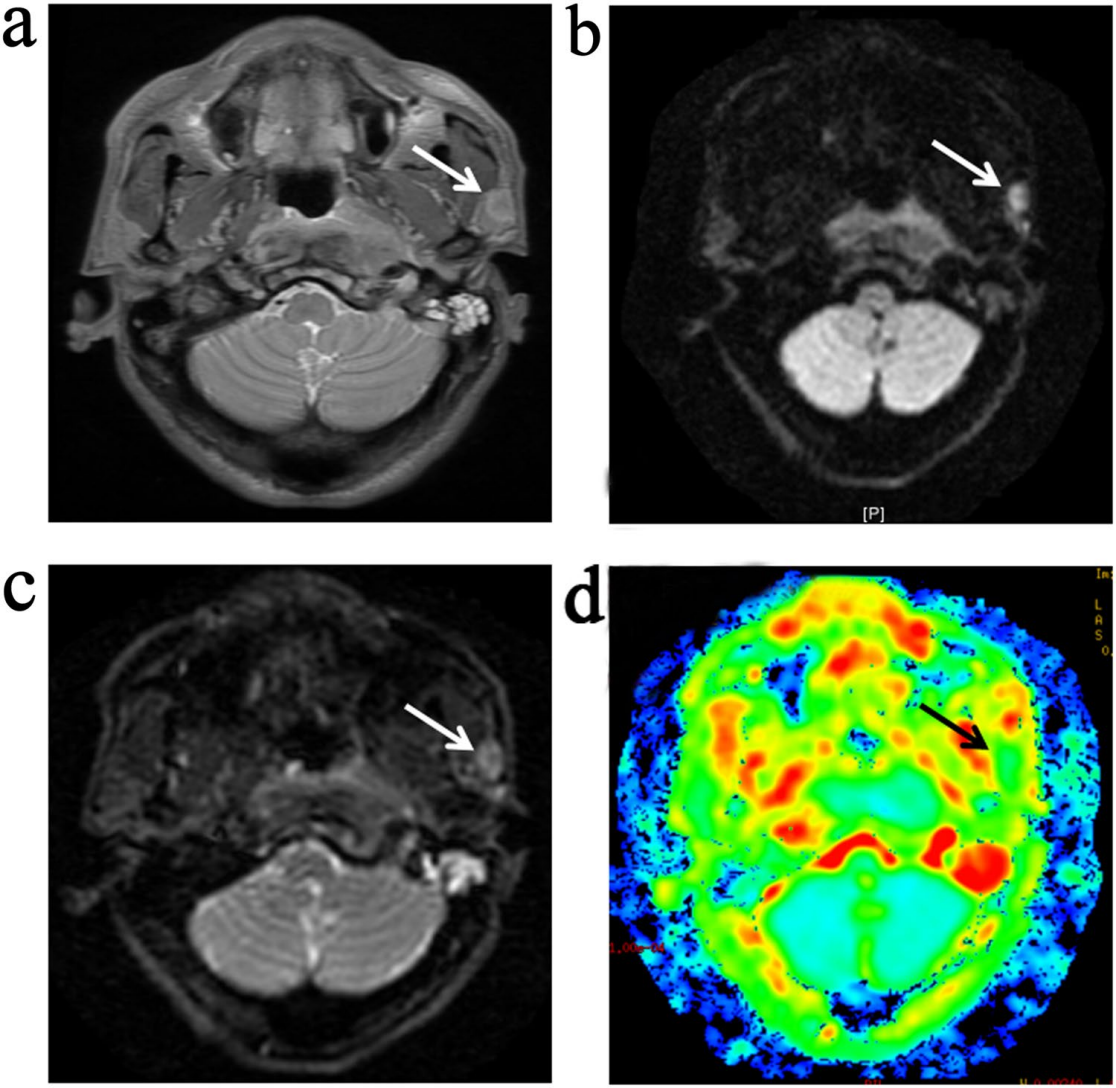
Transverse T2-weighted MR images of a benign PLN (**a**) (arrow). Axial diffusion-weighted MR images of PLN (arrows) show relatively reduced signal intensity achieved at *b* = 0 s/mm^2^ (**b**) and *b* *=* 800 s/mm^2^ (**c**). Consequently, the lesion shows hyperintensity on the ADC map (**d**).

On diffusion-weighted MR images, metastatic PLNs were found to exhibit either homogenous (n = 13) or heterogonous (n = 20) signal intensity. Thirteen metastatic PLNs with homogenous signal intensity showed high signal intensity on MR images performed at *b* value of 0 s/mm^2^ and 800 s/mm^2^, whereas low signal intensity on ADC map (Fig. 1). The remaining metastatic PLNs (n = 20), including necrotic and solid portions, were found to exhibit mixed signal intensity on MR images obtained at two different *b* values, but high signal intensity on ADC map (Fig. 2). The necrotic areas of PLNs presented in the form of bright signals on imaging performed at a *b* value of 0 s/mm, decreased signal intensity at a *b* value of 800 s/mm^2^, while high signal intensity was observed on the ADC map. On the contrary, benign PLNs with reactive hyperplasia exhibited relatively reduced signal intensity at *b* values of 0 s/mm^2^ and 800 s/mm^2^ and appeared hyperintense on ADC maps (Fig. 3).

### ADC values of PLNs and diagnostic ability of DWI

**Figure 4:**
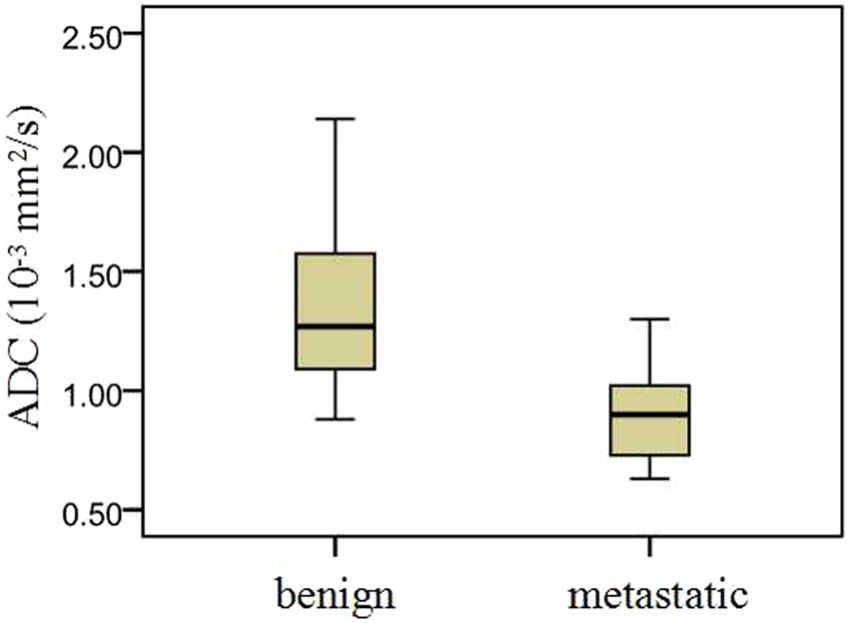
Box and whisker plots demonstrating the ADC values of benign and metastatic PLNs in patients with nasopharyngeal carcinoma. Despite the overlap, the ADC values of the benign PLNs were significantly higher than those of malignant PLNs for NPC (*P* < 0.05).

The mean ADC value of benign PLNs [(1.37 ± 0.17) × 10^−3^ mm^2^/s] was markedly higher than that of malignant PLNs [(0.92 ± 0.36) × 10^−3^ mm^2^/s] for NPC (*P* < 0.05) (Fig. 4). Furthermore, ADC values of PLN metastases ranged from 0.65 × 10^−3^ mm^2^/s to 1.30 × 10^−3^ mm^2^/s, and those of benign PLNs ranged from 0.88 × 10^−3^ mm^2^/s to 2.14 × 10^−3^ mm^2^/s.

**Figure 5:**
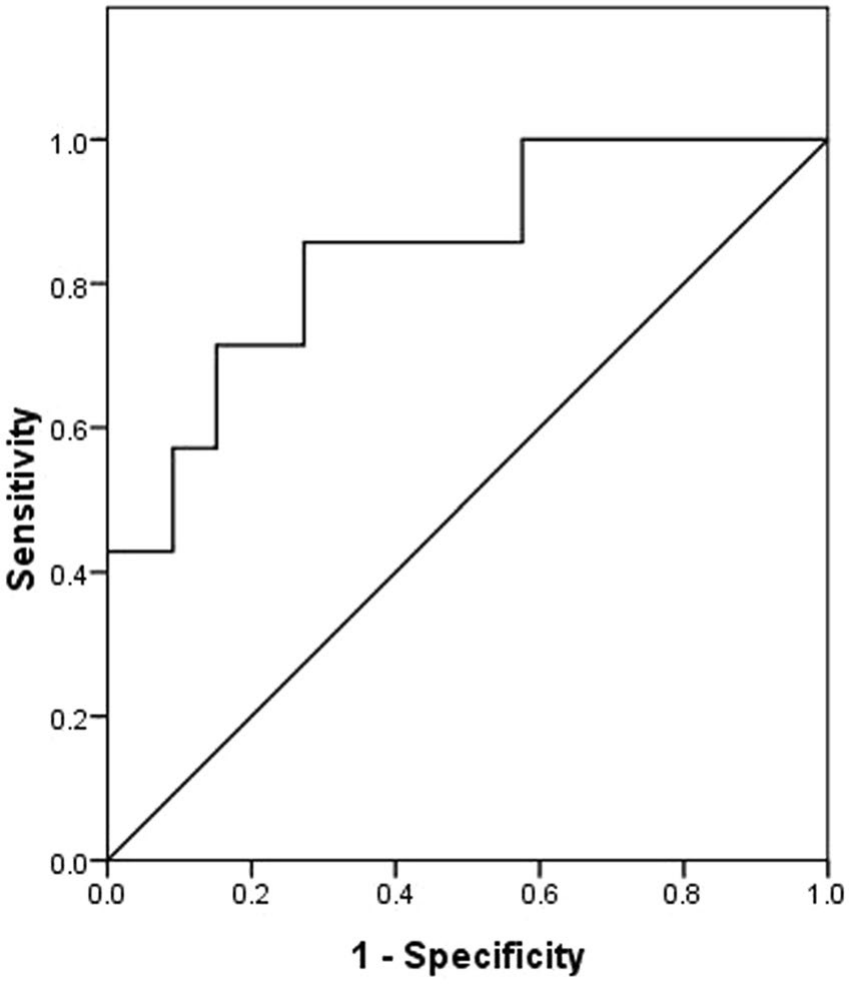
Receiver operating characteristic (ROC) curve analysis carried out for the discrimination between benign and metastatic PLNs by using ADC values. The area under the curve (AUC) is 0.84.

As for the diagnostic ability of DWI in discriminating benign from malignant PLNs, the ROC curve analysis showed that the optimal threshold ADC value for differentiating between benign and malignant PLNs was 1.01 × 10^−3^ mm^2^/s. After applying this cut-off value, the best results could be obtained with a diagnostic sensitivity of 85.7%, a specificity of 72.7%. In addition, the area under the receiver operating characteristic curve (AUC) for the differentiation of benign from malignant PLNs was 0.84 (Fig. 5).

### Consistency between histopathological results and ADC values or MRI features on MRI

The consistency of imaging diagnostic parameters with histopathological diagnosis as the reference standard was examined. As shown in Table 1, a moderate *k* value of 0.483 was found between the reference standard (benign vs. malignant) and the threshold of ADC value (<1.01 × 10^−3^ mm^2^/s vs. ≥1.01 × 10^−3^ mm^2^/s) (*P* < 0.001). However, a fair degree of agreement was observed between the reference standard and the MID (<1 cm vs. ≥1 cm), necrosis (no vs. yes), extranodal extension (no vs. yes), and regional grouping of PLNs (no vs. yes) were all found to be only fair, with a *k* value of 0.257, 0.305, 0.276, and 0.205, respectively.

**Table 1:** Correlation between histopathological diagnoses (reference standard) and the ADC values, the size, and morphological features of 40 PLNs on MRI.

	RS and ADC values (<1.01 × 10^−3^ mm^2^/s vs. ≥1.01 × 10^−3^ mm^2^/s)	RS and MID (<1 cm vs. ≥1 cm)	RS and necrosis (no vs. yes)	RS and extranodal extension (no vs. yes)	RS and regional grouping (no vs. yes)
*k* value	0.483	0.257	0.305	0.276	0.205
*P* value	<0.001	0.084	0.017	0.026	0.033

Abbreviation: RS, reference standard (benign vs. malignant); ADC, apparent diffusion coefficient; MID, minimal axial diameter.

## Discussion

DWI has been reported to effectively differentiate benign from metastatic cervical lymph nodes in patients with head and neck cancers^15,16^. However, to our knowledge, the ability of DWI to discriminate between benign and malignant PLNs has not been investigated due to the relatively low incidence rate of initial PLN metastases or recurrences of NPC^17,18^. In the present study, each PLN was finally characterized by histopathological diagnosis. ADC values of benign PLNs were found to be markedly higher than those of malignant PLNs in NPC. Further, 1.01 × 10^−3^ mm^2^/s was found to be the optimal threshold ADC value to differentiate benign from malignant PLNs, with an overall diagnostic sensitivity of 85.7%, and a specificity of 72.7%. In addition, a moderate degree of agreement was found between the histopathological reference standard and the ADC threshold value, whereas degree of agreement between the reference standard and the size or the morphological features on MRI was observed to be only fair. These findings suggest that DWI may help discriminate benign from malignant PLNs for NPC.

Conventional cross-sectional imaging modalities such as CT and MRI have limited ability to detect malignant infiltration in small sized lymph nodes, whereas augmented lymph nodes may well reflect a reactive pathology instead of a cancerous one^19,20^. However, subtle abnormalities in tissue architecture could be detected with ease by DWI, which is based on the diffusion and displacement of water protons in biological tissues. DWI represents a significant progress in the development of pulse sequences that facilitates a better characterization of pathological processes in the tissues, owing to its distinctive ability to reflect the random extra-, intra- and transcellular motion of water molecules in tissues. In this respect, DWI is able to reflect the tissue microstructure independent from T1 and T2 relaxation^19^. Thus, DWI may make up for the deficiencies of CT and MRI and is a promising diagnostic tool to distinguish between benign and malignant lymph nodes including PLNs.

In order to assess the value of DW-MRI in this respect, we first compared ADC values of PLNs between the two disease groups. We found that benign PLNs manifested significantly higher ADC values than those of malignant PLNs in patients with NPC. Several studies have also reported that metastatic nodes had markedly lower ADC values than benign nodes in head and neck malignancy^12,16,19^, which were in line with our findings. Generally, metastatic lymph nodes tend to have increased cellularity, greater nuclei, higher nucleus-to-cytoplasm ratio, and less extracellular space as compared to that in benign lymph nodes^12,21^, which restricts the water molecular diffusibility in the extra- and intracellular space, with a corresponding decrease in ADC values for PLNs. However, in a study by Sumi *et al*. ADC values of metastatic lymph nodes were found to be significantly higher than those of benign lymph nodes. A possible explanation for this difference may depend on diverse *b* values used in that study. More importantly, they did not analyze the necrotic and solid portions of lymph nodes separately. Necrotic tissue could lead to relatively unhindered diffusibility of water molecules, with resultant high ADC values for metastatic lymph nodes^22,23^. Hence, in our study, necrotic components were excluded from the measurement of ADC values of cancer foci in these PLNs. Nevertheless, further specific analyses of ADC values for necrotic portions of cancer foci in PLNs are necessary.

To further investigate the value of DW-MRI in clinical practice for MR characterization of PLNs, an optimal ADC threshold value should be clearly defined. Our study showed that 1.01 × 10^−3^ mm^2^/s was the optimal threshold ADC value in distinguishing between benign and malignant PLNs, with a diagnostic sensitivity of 85.7%, and a specificity of 72.7%. Consequently, ADC value of less than 1.01 × 10^−3^ mm^2^/s could be used to identify metastatic PLNs in patients with NPC. However, overlap was encountered in a few cases. On one hand, nine histologically confirmed malignant PLNs were found to exhibit a relatively high ADC value and were mistakenly considered to be benign PLNs. This could be explained by the fact that areas of micronecrosis may alter the ADC value in some metastatic PLNs^21^. However, the size of these foci of necrosis may have been smaller than the voxel size on MR images, which was difficult to be identified from MR images. On the other hand, three benign PLNs as evidenced by histology were observed to have relatively low ADC values and were falsely identified as metastatic PLNs. This may be attributable to dense fibrous reaction in these PLNs that may have restricted the diffusion of water protons. According to a histopathological analysis by Razek *et al*., benign lymph nodes tend to have densely packed of fibrotic tissue and dense accumulation of histiocytes, and epithelioid cells^12^. Further research is required to resolve these two problems.

In order to further verify of the optimal threshold ADC value for diagnosis of malignant PLNs, the degree of agreement between histopathological diagnosis and the threshold ADC value was investigated. In addition, nodal size with MID of at least 10 mm, necrosis, extranodal extension, and regional grouping on MR images usually indicate cervical lymph node metastasis in patients with head and neck cancers^21^. Therefore, the degree of agreement between histopathological diagnosis and these diagnostic MRI parameters was also investigated. We observed a moderate degree of agreement between the histopathological diagnosis and use of the optimal threshold of ADC value, while the degree of agreement with other MRI parameters was only fair. This demonstrated that DWI has a higher diagnostic accuracy than separate morphological diagnosis based on MRI findings. As discussed above, the size of lymph node did not seemed to be the most reliable factor for diagnosis of malignant lymph nodes^19,20^. Moreover, occurrence of nodal necrosis in benign conditions is well documented as well^24,25^. Furthermore, MRI diagnosis of lymph nodal extranodal extension is susceptible to subjectivity in routine clinical practice, which may inevitably result in some false-positives. Finally, a fair degree of agreement for histopathological diagnosis with regional grouping of PLNs may be attributed to the limited number of PLNs featured by regional grouping. Taken together, a combination of the conventional MRI morphological criteria and DWI may improve the accuracy of the diagnosis of PLNs.

One of the limitations of the present study was the relatively small cohort of NPC patients who had histologically confirmed benign PLNs, This is because FNAC of PLNs was not performed routinely in our patients unless there was a high suspicion of malignant involvement based on imaging findings. However, this may have affected the accuracy of optimal threshold of ADC value in distinguishing between malignant and benign PLNs. Therefore, further research with a large cohort of benign cases will be needed to validate our results. Moreover, few biopsied PLNs with MID of less than 10 mm were enrolled in our study. In clinical practice, some PLNs are commonly too small to be amenable to FNAC; exploration of noninvasive imaging modalities to identify the nature of small PLNs in NPC appears particularly important. In future, it may be possible to identify even small PLNs as benign or malignant by increasing the number of patients with PLNs of MID of <10 mm. Furthermore, the retrospective study design may have introduced an element of selection bias may exist. Hence, a prospective, multicentre clinical study is required to verify the diagnostic value of DWI technique in differentiating benign from malignant PLNs.

## Conclusion

Diffusion-weighted MR imaging with ADC determination seems to be a promising technique to distinguish between benign and metastatic PLNs in patients with NPC.

## Materials and Methods

### Patients

This retrospective study was approved by the institutional review board at Fujian Cancer Hospital (Ref no. FJCH-09911). Permission was taken to perform the current study with informed consent obtained form each patient. Between September 2008 and August 2016, a cohort of 120 patients with NPC who were newly diagnosed with PLN metastases, or who developed PLN recurrence during follow-up were retrospectively reviewed. Eighty one patients for whom histopathological results were not available were excluded from the study. A total of 39 NPC patients (28 males and 11 females, median age 45 years, age range [11–78 years]) were enrolled, with a combined total of 40 PLNs that were either confirmed by core biopsy (n = 29) or were histopathologically proven after surgical dissection (n = 11). One of these patients had two PLNs (superfical and deep intraparotid, respectively). Fourteen patients were newly diagnosed patients and had never undergone radiotherapy or chemotherapy for the PLNs prior to the histopathological examination. Twenty-five patients developed PLN recurrence; none of these patients exhibited imaging findings suggestive of parotid lymphadenopathy at the time of initial diagnosis. Based on the final histopathological results, 33 PLNs were found to be metastatic nodes and 7 PLNs were diagnosed as benign lymphadenopathy. All patients underwent conventional MRI scans of the nasopharynx and the neck. To ensure that the PLN which received surgical dissection or FNAC was the same PLN as seen at MRI, MRI scans were examined along with the findings on histopathological examination.

### MR imaging

MRI examinations of 30 patients were conducted with a whole-body Multi-Transmit scanner (Achieva 3.0 T TX, Philips Healthcare, Best, Netherlands). Standard 16-channel head and neck array coils were used for both conventional and diffusion-weighted MR imaging to cover PLNs extending from the skull base to the thoracic outlet. The technical parameters of axial T2WI were as follows: repetition time/echo time (TR/TE): 6526/60; 230 × 240 mm field of view (FOV); 5-mm thick section and 1-mm inter-slice gap; number of signal averages (NSA): 2; number of slices: 36; scanning time: 2 minutes 25 seconds. The following parameters were used for diffusion-weighted MR imaging: TR/TE: 4190/69; FOV: 230 × 240 mm; NSA: 2; number of slices: 36; section thickness: 5-mm; interslice gap: 1-mm. All images were obtained by a diffusion factor *b* of 0 or 800 s/mm^2^. The scanning time was 4 minutes 47 seconds. The ADC maps were reconstructed on the workstation (Extended MR Work Space 2.6.3.4, Philips Healthcare) with IDL 6.3 software.

MRI studies of 9 patients were performed with a 1.5-T GE Signa scanner (Signa Excite 1.5 T HD Twinspeed Medical Systems, GE healthcare). Standard 8-channel neurovascular coils (30 cm diameter) were used extending from the temporal lobe to the thoracic outlet with 36 axial slices, field of view of 24 cm and slice thickness of 5 mm with 1 mm gap. Imaging protocol used included axial fat-suppressed proton density–weighted imaging (PDWI) FSE and axial DWI. Diffusion-weighted imaging was conducted with the involvement of a spin-echo/echo planar imaging sequence: the technical parameters of DWI were: repetition time, TR6000 ms; echo time, TE the default minimum; field of view: 24 × 24 cm; NEX: 2; slice thickness: 5 mm; interslice gap: 1 mm; *b* value of 0 and 800 s/mm^2^; scan time 52 seconds^26^. The ADC values were determined on the workstation (GE, Fairfield, Connecticut).

ADC values were determined by two radiologists independently with use of the following equation 1:

$${\rm{ADC}}=(\frac{\mathrm{ln}\,{{\rm{SI}}}_{1}/{{\rm{SI}}}_{2}}{{{\rm{b}}}_{2}-{{\rm{b}}}_{1}})$$

where *b*_1_ of 0 s/mm^2^ and *b*_2_ of 800 s/mm^2^ are representative of gradient parameters of sequences S1 and S2, and SI_1_ and SI_2_ are representative of signal intensity in such sequences, respectively.

### Image analysis

MR images were independently examined by two MRI radiologists who had more than 10 years of diagnostic experience in the context of NPC. Both radiologists were blinded to the final histopathological diagnosis of PLN or clinical stage of NPC. A region of interest (ROI) was manually delineated on the ADC map around the margin of cancer foci in these PLNs with the aid of MR images, and then the ADC value was measured. ADC maps were initiated on the scanner console by the *b* = 0 and *b* = 800 s/mm^2^ images. In general, ROIs were placed manually on the slice with the largest cross-section of the enlarged solid PLNs on imaging of the entire volume; whereas in obvious necrotic lymph nodes, ROIs were only placed manually on the solid tissue portions of PLNs in case of the necrotic components^27^. Necrosis was defined as high signal intensity on T2-weighted images, hypointensity on T1-weighted images, but no enhancement of signal intensity on T1-weighted contrast-enhanced images. If the radiologist detected more than one PLN in MR imaging, it was reasonable to select the largest lymph node as observed in images as compared to the PLN by histopathological diagnosis^27^. The ADC value of each enlarged PLN was used to compare against the histopathological result, which was considered as the reference standard.

### Statistical analysis

All statistical analyses were performed with the statistical software version SPSS17.0 for Windows (SPSS Inc., Chicago, IL). Between-group differences with respect to continuous variables were assessed with Shapiro-Wilk test. The Mann-Whitney *U* test was used to compare the ADC values of metastatic and benign PLNs. Receiver operating characteristic (ROC) curve analysis was performed to assess the diagnostic performance of ADC values to differentiate benign from metastatic PLNs, and to calculate the optimal threshold ADC value which was associated with the highest accuracy in the discrimination of benign from metastatic PLNs. The *k* statistical test was performed to evaluate the degree of agreement between the histopathological diagnoses and the ADC values, the MID, or the morphological features of PLNs. A *k* value of 0.01–0.2 was considered slight agreement, that of 0.21–0.40 represented fair, that of 0.41–0.60 represented moderate, that of 0.61–0.80 represented substantial, and that of 0.81–1.0 represented excellent agreement^28^. A two-tailed *P* value of <0.05 was considered to be statistically significant.
